# On being editor of (Indian) medical journals: A steeple chase

**DOI:** 10.4103/0301-4738.62640

**Published:** 2010

**Authors:** Barun Kumar Nayak, V R Joshi

**Affiliations:** P. D. Hinduja National Hospital and Medical Research Center, Veer Savarkar Marg, Mahim, Mumbai - 400 016, India. E-mail: editor@ijo.in

The itch to become an editor of a Journal exists in but a few. The reasons vary. It could be the honor or the attraction of holding a prestigious position, a position that gives instant visibility, or because of an inherent journalistic tendency, or a desire to contribute to the growth (academic) of the journal, to quote a few. However, editorship is not a bed of roses. Furthermore, editorship carries with it a trust reposed and a responsibility accepted.

By and large there are two types of biomedical publications, one, the non-peer reviewed journals, wherein usually no original research study is published and problems encountered are much less as compared to the peer reviewed journals such as the Indian Journal of Ophthalmology (IJO).

The purpose of peer-reviewed journals is to publish original studies and other articles with the sole aim of the advancement of science. It has to stand up to international competition and has to strictly adhere to publication ethics. Peer-reviewed journals are either publisher owned or association owned (such as IJO) and managed without any major help from any publisher. There is a third category of journals as well, which are owned by non-association organizations such as the Indian Council of Medical Research (ICMR). We will be discussing the challenges faced by the editors of Indian medical journals that are peer-reviewed and owned by an association.

In an ideal world the sole responsibility of an editor should be to screen and edit articles submitted for publication and to publish only genuine science. However, in the real world an Indian editor is a jack of all trades and has to wear many hats, from that of an editor down to that of an attendant. Some of the activities he/she has to indulge in are:

- Organize the editorial office;
- Establish systems for the smooth and efficient running of the editorial office;
- Generate finances;
- Process manuscripts;
- Make decisions;
- Edit articles;
- Copyedit, style edit, proof read;
- Get a reliable, high quality, yet affordable printer;
- Post journals to members and subscribers;
- Take care of the maintenance and constant upgradation of the journal website;
- Subscription management and so on.

This is, not the end of their misery. The big question is QUALITY of the Journal's contents. How does one assure, improve, and maintain it? In the present Indian mindset, the best material is first submitted to a high impact international journal and if not accepted, only then is it submitted to an Indian Journal. Unless our members change this bias, a Journal cannot raise its quality. Members must realize that “*The Journal is of the members, by the members, and for the members*” to paraphrase the famous words of Abraham Lincoln.

It is a fact that there is a lack of knowledge of publication ethics among the Indian authors. This is because there is no formal training of research in the undergraduate as well as postgraduate curriculum.[1] As a basic requirement postgraduate students have to write a thesis based on research, but they are neither supervised nor guided by their guides. Many researchers do not understand the importance of an institutional review board (IRB) / ethical committee approval prior to starting any research. Most view these committees as a nuisance, an obstacle, and a hindrance.

Tenets of Helsinki Declaration[2] are not understood clearly by the authors. They do not understand the implications of plagiarism, duplicate / redundant publication, and multiple submissions.[3] Strict guidelines as per the International Committee of Medical Journal Editors (ICMJE)[4] criteria for authorship are not followed. The issues of guest / gift / ghost authors are rampant.[3] Conflict of interest is not disclosed and the copyright transfer form is signed by authors without reading its contents. There is a general lack of scientific writing skill. These issues get compounded due to factors such as, pressurization of editors by authors for acceptance of their articles, complaints from (disgruntled) members as well as the subscribers.

One important challenge is to generate funds for the journal. The journal is sent free of charge to all members of the association. There are certain disciplines where finances are easy due to industry support. However, for the majority it may be quite difficult. An editor has to learn or possess the skills to generate funds.

Today with each new editor, the editorial office has to relocate leading to problems of finding an adequate office space, appointing and training staff, of providing modern facilities for its efficient and smooth running. There is a lack of professional managing editors who can relieve the editor of routine tasks and allow him to concentrate on policies, innovation, and ensure a high quality of the Journal's contents. Added to this is the fact that most editors are elected. Most lack prior training or experience of working as a member on the editorial board of a peer-reviewed journal.

Finally, most editors are honorary editors. It is not the question of being paid for the job, but it means the editor has to continue with his professional activities for his bread and butter and find (extra) time for the additional responsibility. The result is lesser time to relax, less time for the family (often already so), stress, and its consequences.

Even so most editors do leave their impact on the journal and IJO is no exception. The innovations and initiatives taken in the last five years are given in the appendix. With all the challenges and limitations faced by the Indian medical journals, IJO is ready to pose a stiff challenge to its foreign counterparts.

## Appendix: The innovations and initiatives taken in the last five years by the Indian Journal of Ophthalmology

- Online submission and processing of manuscripts
- Increase in the number of issues per year
- Increase in the international reviewers base
- Open Access policy
- Free full text of all articles since the inception of the journal
- Comfortable financial position
- Indexing with many bibliographic agencies
- Indexing with ISI to get an Impact Factor
- Listing in PubMed Central
- Facility of providing full text of articles from any journal to our members for research
- Ongoing research methodology workshops
- Institution of the (All India Ophthalmological Society) AIOS-IJO award, as an initiative, to increase submission of good research in IJO
